# A Micropowered Chemoresistive Sensor Based on a Thin Alumina Nanoporous Membrane and Sn_x_Bi_k_Mo_y_O_z_ Nanocomposite

**DOI:** 10.3390/s22103640

**Published:** 2022-05-10

**Authors:** Gennady Gorokh, Anna Zakhlebayeva, Igor Taratyn, Andrei Lozovenko, Valery Zhylinski, Michael Iji, Vladimir Fedosenko, Abdelhafed Taleb

**Affiliations:** 1 R&D Laboratory of Nanotechnologies, Belarusian State University of Informatics and Radioelectronics, 220013 Minsk, Belarus; gorokh@bsuir.by (G.G.); zakhlebayeva@bsuir.by (A.Z.); lozovenko@bsuir.by (A.L.); ijiolakunle2002@gmail.com (M.I.); v.fedosenko@bsuir.by (V.F.); 2 Instrumentation Engineering Faculty, Micro- and Nanotechnology Department, Belarusian National Technical University, 220013 Minsk, Belarus; mnt@bntu.by; 3 Department of Chemistry, Technology of Electrochemical Production and Electronic Materials, Belarusian State Technological University, 220006 Minsk, Belarus; zhilinski@yandex.by; 4 Institut de Recherche de Chimie Paris, Chimie ParisTech, PSL University—CNRS, 75005 Paris, France; 5 Sorbonne Université, 75231 Paris, France

**Keywords:** semiconductor metal oxide composites, micropower gas sensor, nanoporous anodic alumina matrixes

## Abstract

This work presents and discusses the design of an efficient gas sensor, as well as the technological process of its fabrication. The optimal dimensions of the different sensor elements including their deformation were determined considering the geometric modeling and the calculated moduli of the elasticity and thermal conductivity coefficients. Multicomponent Sn_x_Bi_k_Mo_y_O_z_ thin films were prepared by ionic layering on an anodic alumina membrane and were used as gas-sensitive layers in the sensor design. The resistance of the Sn_x_Bi_k_Mo_y_O_z_ nanostructured film at temperatures up to 150 °C exceeded 10^6^ Ohm but decreased to 10^4^ Ohm at 550 °C in air. The sensitivity of the Sn_x_Bi_k_Mo_y_O_z_ composite to concentrations of 5 and 40 ppm H_2_ at 250 °C (10 mW) was determined to be 0.22 and 0.40, respectively.

## 1. Introduction

The rapid and perpetual development of the technosphere, to meet the needs of industrial activity, is at the origin of risks for human health and the balance of the ecosystem. To counter this risk, permanent monitoring and control of our environment is required through the development of reliable and efficient tools [1]. Sensors are the main technical means for this monitoring, and their number is rapidly increasing, as are the requirements for their functional characteristics [2]. The last two decades have seen an intense and continuous development of research in the field of chemical sensors for the detection of different toxic gases, which allows the monitoring of toxicity levels. Priority has been given to the development of new types of chemical sensors to anticipate risk and, thus, measure very small concentrations of chemical pollutants in the environment [3]. Among the environmental pollutants is CO gas. For the detection of this gas, chemical sensors have shown a very high efficiency, especially the so-called resistive sensors, consisting of an active layer of a film of semiconductor material, whose resistance varies in the presence of a certain type of gas. The new classes of sensors are subject to increased performance requirements, such as sensitivity to trace gases, selectivity in analyzing complex gas mixtures, miniaturization of the device, minimization of power consumption, and low cost [4]. These ever-increasing demands require the development of innovative materials with new properties, as well as the use of new design approaches and preparation methods [5,6,7]. The improvement of the sensitivity of the sensors and, thus, the detection of gases at extremely low concentrations requires the increase in the specific surface of the gas-sensitive layer [7,8,9], and the choice of material sensitive enough and suitable for the operating temperatures of the sensor [10,11,12]. To increase the selectivity of sensors, metal oxide compounds of complex composition have been used in the formation of a sensitive layer in the form of nanopowders or nanostructured films [13,14]. Metal oxide nanomaterials were used in gas sensing for the detection of various explosive and toxic gases such as (ethanol, toluene, formaldehyde, CH_4_, CO, NO_2_, O_3_, H_2_S, etc.) [15,16]. Sensors based on (Pd, Fe)-modified SnO_2_ and Pt-loaded SnO_2_ showed better selectivity towards CO and H_2_, respectively, when exposed to a mixture of the two gases [17]. Furthermore, the SnO_2_-PdO_x_-based sensors were found to be capable of selectively detecting both single-component systems, such as hydrogen in air and carbon monoxide in air, and two-component systems, such as the mixture of both gases in air [18]. Although these nanomaterials increase the specific surface area in contact with the gas, improve the selectivity and reduce the response time, they lead to an increase in heat loss and, consequently, an increase in the energy consumption of the sensor [19]. In addition, metal oxide gas sensors, whose operation is generally based on surface or bulk conductance effects, have additional disadvantages, including the need for high-temperature activation steps, cross-sensitivity, and limited stability due to variations in humidity and chemisorbed O_2_ [20]. Most of these sensors have been prepared on silicon substrates as microelectromechanical systems (MEMS), using microtechnology techniques. The sensor structure is based on two thin dielectric layers of silicon oxide and nitride. These thin dielectric layers ensure fast sorption, minimize the response time of the sensor, and minimize the heat loss when heating the semiconducting metal oxide layer, depending on the type of gas detected, up to 250–500 °C [21,22,23,24].

However, MEMS-silicon technology suffers from some important drawbacks. Among them, there is the labor intensity and exorbitant cost of labor, which makes the profitability very low. There is also the mismatch in the thermal and mechanical properties of the thin films used in mono- and polysilicon, silicon oxide, and nitride, as well as platinum and tin oxide, which leads to weakened performance and decreased reliability of the sensors [25]. In addition, the adhesion of the platinum film to the silicon oxide and nitride films needs to be improved. Thus, in practice, it appears that the improvement of some properties is at the expense of others, so the optimization and improvement of sensor performance by increasing reliability and reducing cost remain a rather open research topic [26,27].

In recent years, a new approach has emerged to overcome the problems of MEMS-silicon technology, allowing the fabrication of sensors with improved characteristics. This method is associated with the deposition of the gas-sensitive layer on a nanoporous template [28,29,30].

The method using nanoporous matrices has been shown to be very effective for the preparation of nanostructured films. The nanoporous anodic alumina (AA) matrix is one of the most widely used and has a very compact hexagonal structure with hollow channels in the center. Nanoporous AA matrices have multiple advantages, the main one being their regular and well-ordered nanostructure, with a wide range of pore diameters (from 10 nm to 150 nm). In general, the alumina matrix is also characterized by a uniform layer with a large specific surface area, and interesting mechanical, electrical, and optical properties. The active layers deposited on the nanoporous AA matrix provide the sensor with a large specific surface area and, therefore, a high sensitivity [31,32,33]. In addition, the multicomponent nanostructured active layers deposited on a nanoporous template improve the selectivity of the sensors towards gases with complex composition and their mixtures [34,35,36,37]. The effective use of anodic alumina as a thin film platform to create sensors for various purposes has been reported by many authors in the literature [38,39,40].

The use of nanoporous AA as substrates with high mechanical properties [41,42] in the fabrication of gas sensors significantly reduces their energy losses during operation. As shown by theoretical calculations and experimental data, the energy consumption of the sensors decreases with increasing substrate porosity [28,29]. Anodic alumina substrates have great potential for the development of chemical sensors based on micropowered semiconductor materials. The combination of a thin AA membrane embedded in a silicon wafer has all the advantages of the nanoporous structure in this type of design but does not achieve the high workload of MEMS-silicon technology. The analysis of the creation of gas sensors shows that minimizing the geometrical dimensions of the sensor by using a thin AA nanoporous membrane, on which a thin submicron layer of a mixture of several oxides has been deposited, allows a significant reduction in the energy consumption of thin film chemical sensors, without deteriorating their performances. The power consumption problem limits the creation of multicrystal microsystems for the analysis of gas mixtures of complex composition, as well as the development of low-cost multifunctional portable gas sensors.

In this work, we have demonstrated the possibility of fabricating chemical gas sensors on thin AA membranes, which have many advantages over those fabricated using silicon MEMS technology, particularly in terms of power consumption, sensitivity, reaction rate and regeneration. The original design of a high performance chemoresistive gas sensor, using the profiled AA template and the ionic layering technology for a deposition of a multicomponent nanostructured oxide layer, has been developed.

## 2. Materials and Methods

### 2.1. Fabrication of Anodic Alumina Substrates with Thin Membranes

For the fabrication of anodized substrates with anodic alumina membranes, an aluminum foil (99.99%) with a thickness of d = 100 μm and a size of 60 × 48 mm^2^ was used. To remove mechanical stresses, as well as contaminants and microroughness from the foil surface, the samples were subjected to thermal leveling under a pressure of about 10^7^ Pa at a temperature of 450 °C and to chemical cleaning.

The process of preparing aluminum foil blanks for substrate production was carried out in several steps. First, 60 × 48 mm^2^ plates were cut from 100 μm thick aluminum foil, which was washed in a solution of a chromium mixture and, then, washed with distilled water to remove organic contaminants. Then, compaction of the aluminum blanks was carried out. In the volume of the rolled aluminum foil, there are microscopic cavities and voids, which are the cause of the formation of an irregular porous structure during anodizing. Therefore, the parts were subjected to the multiple rolling process through polished rollers. Following repeated rolling through polished rollers impregnated with a purified kerosene-based rolling compound, the sheet was compacted, and the surface was mechanically polished. The polished plates (more than 10 pieces) without washing were moved with parchment and placed between two 8 mm thick polished steel plates and pressed with a mechanical press under a pressure of about 10^7^ Pa, after which they were fixed with fixing screws. The entire structure was placed in a muffle furnace, heated to 450 °C, held at this temperature for two hours, and removed from the furnace after the blanks had cooled completely to room temperature. This procedure eliminated mechanical stresses in the foil after the mechanical treatment, and the foil was not deformed during anodizing.

Subsequently, the samples were electrochemically polished in an aqueous solution of chromic anhydride 185 g/L and orthophosphoric acid 1480 g/L at a temperature of 353 K in pulsed mode [28] with a pulse duration of 5 s and a duty cycle of 35 s. The total processing time (the sum of the pulse durations) was 10 min at a current density of 650 mA/cm^2^. After electrochemical polishing, the samples were thoroughly washed in distilled water and dried in a centrifuge and in a thermostat at 120 °C for 30 min.

The operations described were carried out in the technological section of the micromechanics department of the Joint-stock company (JSC) “Minsk Research Institute of Radiomaterials” (Minsk, Belarus), a scientific and technological research establishment. All tools and mechanical assemblies were made according to the drawings developed in PLANAR (Minsk, Belarus). Planar JSC (KBTEM) is a scientific and technical group of specialized designers, technicians, and production engineers, which develops and supplies special systems for the realization of critical microelectronic technologies.

The foil was anodized in a 0.5 M oxalic acid electrolyte at a temperature of 14–15 °C, at an anodizing potential of 55 V and an anodizing current of no more than 16 mA/cm^2^ for 75 min. During the anodization process, the electrolyte was stirred with a thermostatically controlled electromagnetic stirrer to eliminate the temperature gradient throughout the electrolyte volume, and to obtain a high quality ordered nanostructured porous aluminum oxide, without local weft zones or thickness spreading. In situ recording and monitoring of the electrical process parameters was performed using a general-purpose interface bus (GPIB, IEEE 488) connected to a personal computer running Keysight BenchVue software, version 2018, Santa Rosa, CA, USA.

**Figure 1:**
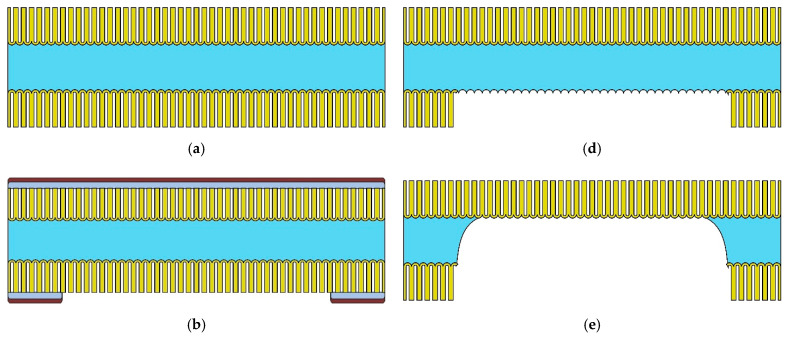

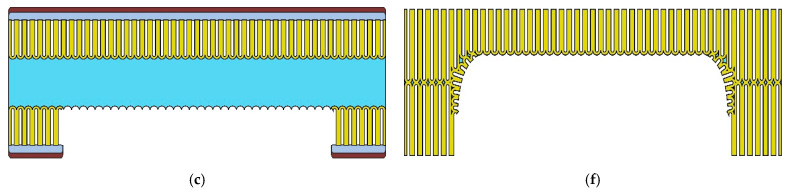
Schematic route for the manufacture of profiled substrates with thin membranes. (**a**) Two-sided anodization in 0.5 M oxalic acid under anodic voltage 55 V. (**b**) Coating with a protective vanadium layer and photoresistive mask, photolithography. (**c**) Selective etching of porous AA in windows of the photoresist mask. (**d**) Removing of photoresist mask. (**e**) Selective chemical etching of aluminum interlayer in the windows. (**f**) Final two-sided through anodization of profiled substrate in 0.5 M oxalic acid under anodic voltage 55 V.

Figure 1 shows a schematic pathway for the manufacture of the thin membrane profile substrates. After the first anodizing step, a 20 μm thick AA layer was formed on both sides of the sheet (Figure 1a). Then, a 200 nm thick vanadium protective layer was applied to the part on both sides by magnetron sputtering, and a photoresist mask with windows was formed on one side to create depressions in the substrate. The size of the windows corresponded to the size of the active part of the sensor (Figure 1b). Vanadium was selectively removed in a 30% hydrogen peroxide solution in the open windows during masking until the aluminum oxide surface. Then the porous AA was dissolved in the windows in a selective solution of 60 g/L H_3_PO_4_ and 20 g/L CrO_3_, the photoresist mask in a mixture of monoethanolamine and dimethylformamide in a ratio of 1:9, and the vanadium in a 30% hydrogen peroxide solution (Figure 1c). Then, the operation of the selective dissolution of aluminum in the windows was carried out over the whole thickness of porous AA in a solution of 1.25 g/L CuCl_2_, 1L HCl, 158 mL/L H_2_O (Figure 1e), followed by chemical purification in 90% HNO_3_. In the final stage, bilateral anodizing of the resulting profiled substrate was carried out under conditions corresponding to the first anodizing step (0.5 M oxalic acid, temperature 14–15° C, voltage 55–56 V). Under these conditions, the samples were kept until the aluminum had oxidized in depth for 100–110 min, while a decrease in the anode current to almost zero was observed. During the anodization process, the electrolyte was continuously stirred using a thermostatically controlled electromagnetic stirrer. As a result of the technological operations carried out, a profiled AA substrate with thin membranes formed on it to create active thin film gas sensor structures was obtained (Figure 1f).

### 2.2. Development of Gas Sensor Design on the Thin Membrane in the Anodic Alumina Substrate

When creating sensor structures on profiled AA substrates, the optimal structural parameters were initially determined considering the thermomechanical characteristics of the thin membranes in the substrate, as well as the thermal conductivity characteristics in the perpendicular and tangential directions with respect to the surface [43,44]. Based on the previously obtained calculated data [28], temperature distribution models in membranes of different sizes were compiled, taking into account their porosity and the influence of their size on the energy consumption of the sensor, to keep the gas-sensitive layer within the operating temperature range of 200–500 °C. Based on the calculations, the design was developed and the optimal dimensions of the structural elements of the chemical sensor were determined, which ensured the minimum power consumption while maintaining all the mechanical properties of the substrates: the sensor area on the AA substrate was 1.5 × 1.5 mm^2^, the heater area was 450 × 450 μm^2^, the width of the external electrodes was 50 μm, the distance between the contact areas was 0.7 × 0.7 mm^2^, the size of the contact areas was 300 × 300 μm^2^. Such geometrical dimensions should ensure the limitation of the temperature field distribution over the membrane in the substrate and localize heat in the heater region and prevent its propagation along the platinum heater on its outer perimeter [23,28].

**Figure 2:**
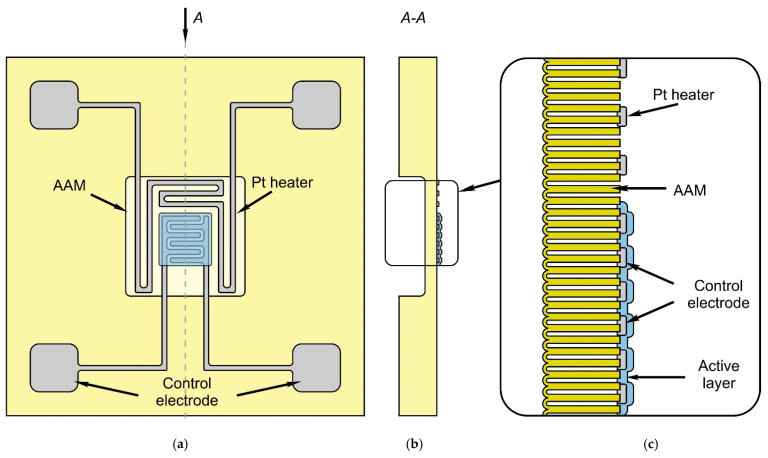
Schematic representation of a gas sensor based on a profiled substrate with a thin AA membrane (**a**); schematic cross-sectional view of the sensor in the middle of the membrane (**b**); design of a platinum heating element covered with a SiO_2_/Si_3_N_4_ dielectric bilayer (**b**); enlarged image of the insert with detailed cross-sectional view of the sensor elements (**c**).

Figure 2 shows the schematic of the developed thin-film sensor based on the profiled substrate with thin membranes on the surface. The gas sensor consisted of an AA substrate with overall dimensions of 1.5 × 1.5 × 0.1 mm^3^, in the center of which the dielectric AA membrane of 500 × 500 × 20 μm^3^ was formed. A platinum meander heater with three sections and an electrode thickness of 0.45 μm was located along the perimeter of the membrane (Figure 2a). In the center of the membrane was a measuring element in the form of an interdigital capacitor, consisting of three pairs of electrodes 140 µm long, 20 µm wide, and with a gap of 15 µm between them. The gas-sensitive layer (Figure 2b) of Sn_x_Bi_k_Mo_y_O_z_ was deposited on top of the electrodes by the ion layering technique (Figure 2c) [45,46,47,48,49,50].

### 2.3. Fabrication of Gas Sensors on the Thin Membranes in the Anodic Alumina Substrate

**Figure 3:**
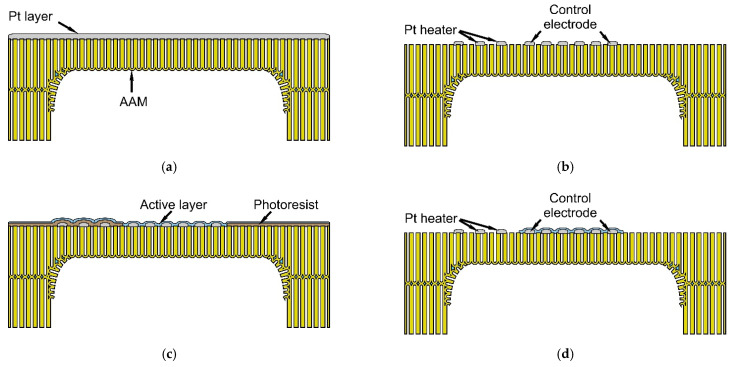
Schematic steps for the manufacture of the profiled substrates with thin membranes. (**a**) Magnetron sputter deposition of platinum layer of thickness 0.45 µm. (**b**) Ion-beam etching of platinum and formation on heater topology. (**c**) Coating of double dielectric layer SiO_2_/Si_3_N_4_ and ionic layering of multicomponent gas-sensitive layer of Sn_x_Bi_k_Mo_y_O_z_. (**d**) Formation of system measuring electrodes.

A chemical sensor based on an AA substrate was prepared as follows (Figure 3). A 0.45 µm thick platinum thin film was deposited on a 100 µm thick AA substrate by magnetron sputtering (Figure 2a). Then the following steps were performed: first, we performed photolithography and ion-beam etching of the platinum to form the desired topology on its planar side in terms of meander heater with electrodes and interdigital measuring element with external contact pads (Figure 3b). Then, through the mask, the area of the interdigitated measuring element was covered by the multicomponent gas sensitive layer of Sn_x_Bi_k_Mo_y_O_z_ after 20 cycles of cyclic ionic layering of the matrices in cationic and anionic solutions with intermediate washes in distilled water (Figure 3c) [51,52,53]. Prior to ionic layering, the matrices were held in distilled water at *T* = 360–365 K for *t* = 30 min. The multicomponent composite Sn_x_Bi_k_Mo_y_O_z_ was formed by alternating two cationic and anionic solutions. In the first cycle, the aqueous solution of SnCl_2_ × 2H_2_O (pH = 1.35) was used as the cationic solution and the aqueous solution of (NH_4_) 2MoO_4_ (pH = 2.45) as the anionic solution. In the second cycle, an aqueous solution of BiCl_3_ × 2H_2_O (pH = 1.65) was used as the cationic solution and the aqueous solution of (NH_4_) 2MoO_4_ (pH = 2.45) as the anionic solution. Each cycle involved treatment of the matrix in a cationic and anionic solution with intermediate washes in distilled water. The first and second complete cycles were repeated 10 times, and metal oxide films of 20 monolayers thick were formed, in which a monolayer of Sn_x_Mo_y_O_z_ alternated with a monolayer of Bi_k_Mo_y_O_z_. The concentration of the solutions was 0.1 M, and the temperature was *T* = 20 ± 2 °C.

Then, the mask was removed, and the whole system was annealed for 30 min at a temperature of 480 °C in an argon atmosphere, to create a reliable contact with the platinum electrodes and form the required microstructure. The thickness of the active layer after drying did not exceed 1.0 µm on the surface of the heating element. In the final stage, the system of measuring electrodes and external contact pads was developed (Figure 3d).

### 2.4. Methods for Studying the Microstructure, Composition, and Properties of Thin-Film Systems

The morphology of the AA matrices with the deposited composite films, as well as the cross sections of the samples, were studied using a Hitachi S-806 scanning electron microscope (SEM) at an accelerating voltage of 10–15 kV and a magnification of (15–40) × 10^3^ times.

X-ray electron probe spectral microanalysis of AA/(Sn_x_Bi_k_Mo_y_O_z_) was performed using the “Bruker” QUANTAX 200 add-on with the XFlash^®^6 Silicon Drift Detector (SDD) 60 mm^2^ active area for scanning electron microscopy. The energy resolution was <125 eV at Mn-Kα, detection from Beryllium (Z = 4) to Americium (Z = 95). The primary beam spot of energy 15.4 keV had a characteristic size of 2 μm and was directed towards the cross section of the cleaved samples. The penetration depth of the beam was from 0.1 μm to several microns.

The response of the microsensors to pure gases and their mixtures was carried out using a mass flow measurement system consisting of a PC and computer-controlled mass flow controllers. The mass flow meters had a full-scale resolution of 1%. Commercially available calibrated gas cylinders were used for the experiments. The mass flow controllers were calibrated with synthetic air. This did not lead to significant errors, as the experiments were performed with target gases highly diluted in air. The volume of the test chamber was 36 cm^3^. The total flow rate was adjusted to 100 cm^3^/min. The sensor characterization was performed by DC resistance measurements. The electronic measurement system consisted of a Keithley Instruments Inc. electrometer (model 6517A) with a data acquisition card (model 6522), which provided ten channels for measuring the active layer resistance. Measurements of active layer resistance for each operating temperature and target gas at different concentrations were repeated 5 times to determine the repeatability of the sensor response.

## 3. Research and Discussion

### 3.1. Electron Microscopic Studies of Profiled Anodic Alumina Substrates

**Figure 4:**
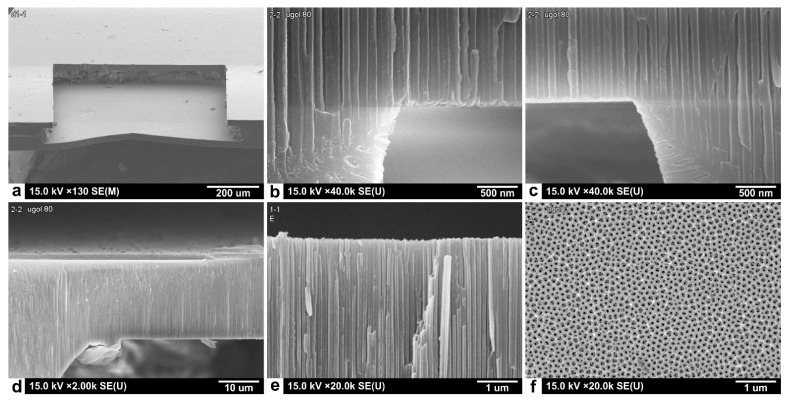
SEM images of the AA substrate with thin membranes: (**a**) image of the membrane from the back side of the substrate; (**b**,**c**) images of the transitions at the membrane–substrate interface; (**d**) image of the general view of the membrane cross-section; (**e**) image showing the microstructure of the porous AA substrate; and (**f**) image showing the surface of the anodic alumina membrane.

The technological route described in Section 2.1 was used to manufacture thin film AA substrates. Figure 4 shows electron microscope images of various elements of the profiled substrates and the microstructure of the anodized alumina on these substrates. Figure 4a shows the image of the reverse side of the profiled substrate with the thin membrane. The membrane had a thickness of 20 μm and a size of 0.50 × 0.50 mm^2^ (Figure 4a,d).

As can be seen in Figure 4, the AA membranes had a regular profile, the same thickness, and met the flatness requirements. The electrochemical anodization process resulted in the porous AA layer of uniform thickness on the substrate and the same for all the membranes. The double-sided anodization process resulted in membranes with a uniform profile and avoided the occurrence of stresses at the membrane/substrate interface and local defects along the substrate surface. Figure 4b,c show transitions at the membrane–substrate interface. The smooth profile of the membrane–substrate interface, formed by the tangential (lateral) anodization, smoothed the stresses on the edges of the membranes and excluded possible deformation phenomena. Figure 4e shows the general view of the membrane cross section, and it can be observed that the pore diameter of the substrate and the membranes did not exceed 40 nm, and the pores were uniform and regular (Figure 4e,f). The developed method allows the formation of membranes with a thickness of 5–30 μm and a surface area of up to 2 mm^2^ in AA substrates with a thickness of 80–150 μm. The pore diameter of the AA was in the range of 30–50 nm, and the porosity of the aluminum oxide did not exceed 20%.

### 3.2. Study of Multicomponent Gas-Sensitive Layers of Sensor on Nanoporous Substrate

**Figure 5:**
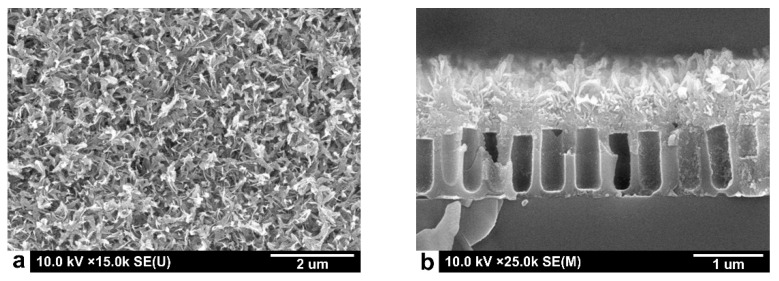
SEM images of the Sn_x_Bi_k_Mo_y_O_z_ gas sensitive layer deposited on the anodized alumina matrix, (**a**) front view and (**b**) profile view.

SEM images of the gas sensitive Sn_x_Bi_k_Mo_y_O_z_ thin films deposited on the AA membrane (AA matrix) are shown in Figure 5. As can be seen from these SEM images (Figure 5a), the Sn_x_Bi_k_Mo_y_O_z_ thin films were formed by nanosheets with a thickness of about 50 nm and randomly oriented (Figure 5b).

**Figure 6:**
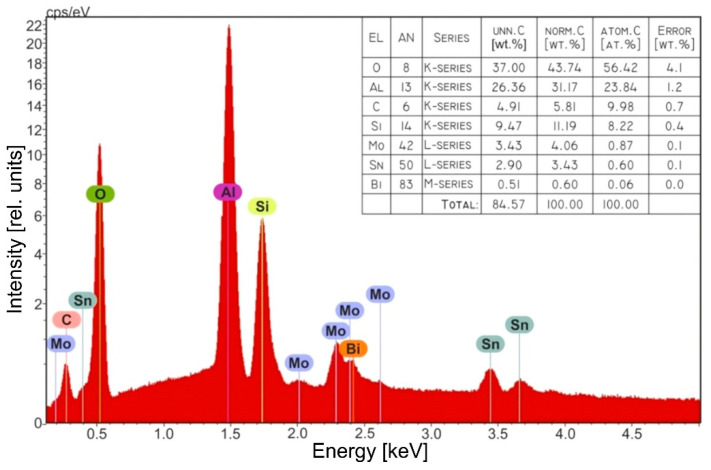
EDX results of prepared AA/(Sn_x_Bi_k_Mo_y_O_z_) structure; the insert table presents the analysis details.

The results of the elemental composition analysis of the formed structures by energy dispersive X-ray spectroscopy (EDX) are presented in Figure 6. The EDX spectrum of the formed structures contained peaks characteristic of the matrix used (Si-1.72 eV; Al-1.48 eV; O-0.52 eV) and the thin film formed on the matrix (Sn-0.4, 3.42, 3.68 (max); Mo-0.2, 2.01, 2.3 (max), 2.4, 2.61; O-0.52 eV). The maximum tin peak shifted from 3.42 to 3.68. The atomic ratio of tin, molybdenum, and bismuth in the formed AA/Sn_x_Bi_k_Mo_y_O_z_ structure was Sn:Bi:Mo = 0.60:0.06:0.87 at%.

The formed Sn_x_Bi_k_Mo_y_O_z_ films consisted mainly of Sn-Mo oxides with Bi dopes, and the molybdenum content predominated in the film.

The gas-sensitive composites based on the deposited Sn-O/Bi-O/Mo-O oxides had a crystalline structure and consisted of interconnected nanosheets with a large specific surface area. These nanostructured thin films of n-type semiconductors interact with reducing gases (e.g., hydrogen, CO, ethanol and other alcohols). The oxidation of the adsorbed gas molecules results in the enrichment of the semiconductor conduction band with electrons, the decrease in the potential barrier, and, consequently, the decrease in the resistance of the metal oxide composite films [54].

### 3.3. Investigation of the Thermomechanical Characteristics of Chemoresistive Sensors on a Nanoporous Anodic Alumina Membrane

When designing and fabricating a chemoresistive sensor based on a nanoporous membrane on an AA substrate, it is important to have reliable data on the thermomechanical parameters of the individual sensor elements during repeated heating and cooling, as well as on the temperature distribution (heat losses) during sensor operation. This section presents analysis of the dependences for the elastic moduli and thermal conductivity coefficients obtained based on modeling by the finite element method using the standard and experimental methodology reported in the literature [28]. To determine the thermomechanical properties of a nanoporous AA with a regular structure, the finite element method [45] was used using the COMSOL Multiphysics 3.5a finite element simulation package. The determination of the coefficients of the longitudinal and transverse thermal conductivity of the porous material was achieved using imitation modeling [23].

The data reported in [28] were used to evaluate the optimal energy consumption for the design of AA membrane-based chemical sensors, as shown in Figure 2. The objective was to evaluate the energy consumption of the sensor by limiting the substrate heating to the temperatures required for sensor operation (300–550 °C). To consider the heat loss factor at the lead wires of the metallization contacts, the temperature was set at 120 °C. It should be noted that the temperature field remained localized in the region of the platinum electrodes, with an increase in the porosity up to 20%, and beyond that, this effect was enhanced. Furthermore, the influence of the membrane thickness on the energy consumption of the sensor was studied, i.e., the power of the electric current, which was spent to maintain the gas-sensitive layer at temperatures in the range of 500 °C to 550 °C.

**Figure 7:**
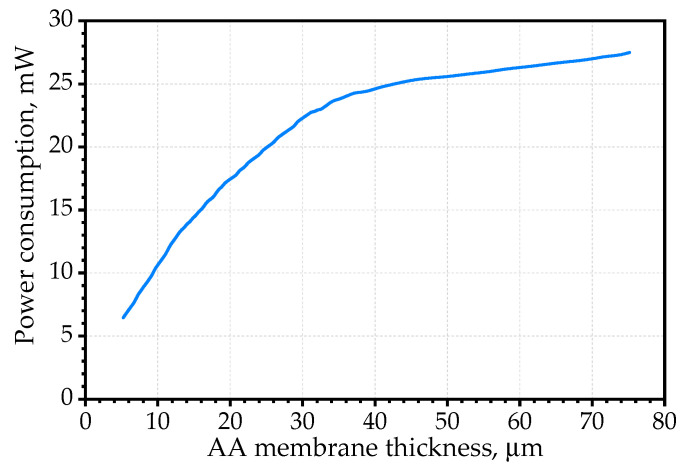
Dependence of the power consumption on the thickness of the gas-sensitive layer membrane with a porosity of about 20% at temperature of 500 °C.

The calculated dependence of the energy consumption of the gas-sensitive layer on the membrane thickness of 20% porosity at temperature of 500 °C is shown in Figure 7. It is obvious that with the increasing membrane thickness the energy consumption for heating increased steadily; however, from the membrane thickness of 40 μm, the energy dissipation became significant, and the membrane thickness no longer had a significant effect.

**Figure 8:**
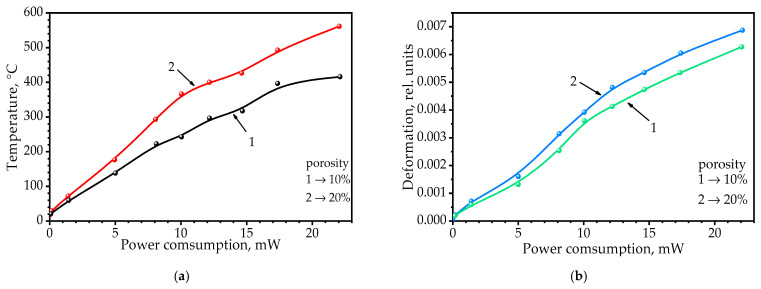
(**a**) Dependence of the heating of the gas-sensitive layer on the power dissipated by the heating element for a membrane porosity of 10% (curve 1) and 20% (curve 2). (**b**) Dependence of the membrane deformation on the power dissipated by the heating element for a membrane porosity of 10% (curve 1) and 20% (curve 2).

When predicting the reliability of the sensor operation, the ability of the sensor to resist thermomechanical deformations occurring during its operation at elevated temperatures was considered. Figure 8a shows the calculated values of the temperature of the sensor’s sensitive layer, and Figure 8b shows the magnitude of the relative thermomechanical deformations that occurred there as a function of the sensor’s power consumption. The membrane with higher porosity heated up faster and reached higher temperatures and lower dissipation power (Figure 8a, curve 2). It was found that porosity had no significant effect on thermomechanical deformations.

In the AA membrane with a volumetric porosity of ~20% (Figure 8b, curve 2), the value of the thermomechanical deformations that occurred was only 10% higher than that of the membrane with a volumetric porosity of ~10% for a sensor power consumption of 23 mW. Calculations have shown that at a voltage higher than two volts applied to the heating element, thermomechanical deformations higher than 0.01 of the membrane occur, which is a critical value for this type of membrane.

### 3.4. Measurements of the Electrophysical Parameters of Sensor Based on Nanoporous Substrate

The sensors based on AA dielectric membranes, manufactured according to the method described above, were tested in the measurement of H_2_ and CO. The response was analyzed at power consumption values between 5 and 22 mW, which allowed the sensitive element to be heated to a temperature between 150 °C and 550 °C. A detailed measurement procedure was described in Section 2.4. The sensors were exposed to each gas concentration for 5 min; then, air was used to purge the measurement platform for 30 min. The sensor response was defined as S = R_gas_/R_air_ in the case of oxidizing and reducing gases, where R_air_ is the sensor resistance in air (steady state), and R_gas_ is the sensor resistance after 5 min of gas exposure.

**Figure 9:**
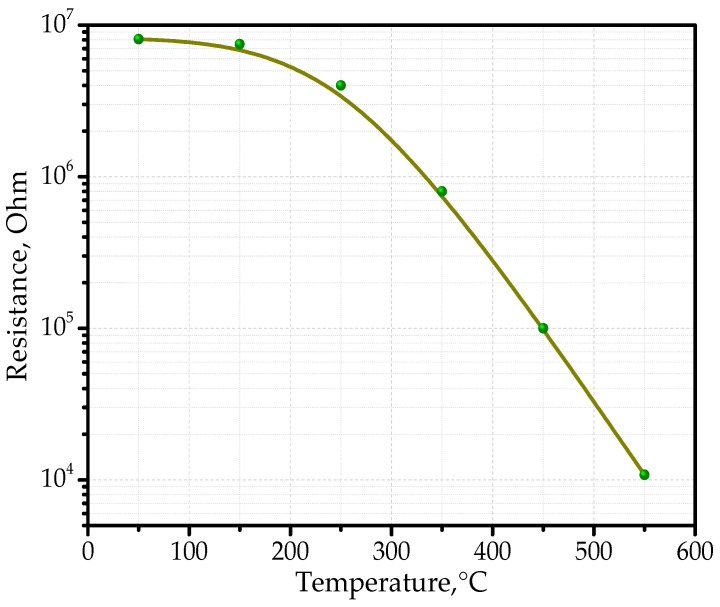
Curve showing the variation of the resistance of the Sn_x_Bi_k_Mo_y_O_z_ sensitive layer to gas in air as a function of temperature.

To determine the sensor maximum sensitivity, we studied its dependence on the power supplied to the sensor heating element at a certain concentration of the gas mixture. In this case, the sensitivity obtained should be related only to this concentration; the magnitude of the sensor response at other concentrations may be different. Generally, the maximum sensitivity is determined for a certain temperature range corresponding to the selected concentration of the gas mixture. For each of the provided powers, the resistances of the sensitive layer were measured in air; then, the chamber was purged with the tested gas mixture, and after the equilibrium state of the gas mixture was established in the chamber, the resistance values were measured. At the same time, the response time (the time between the beginning of the resistance change and its steady state) and the relaxation time of the layer resistivity to the initial value after the end of the feeding of the chamber with the gas mixture, were recorded. These parameters are important for establishing the practical modes of operation of the sensors. Figure 9 shows the experimental dependence of the variation of the resistance of the gas sensitive Sn_x_Bi_k_Mo_y_O_z_ layer in air on temperature.

In terms of resistance, the metal oxide composite Sn_x_Bi_k_Mo_y_O_z_ can be classified as a high resistance semiconductor. The resistance of Sn_x_Bi_k_Mo_y_O_z_ thin films at temperatures up to 150 °C was around 10^6^ Ohm, but with the subsequent increase in temperature, a monotonic decrease, close to linearity in the logarithmic scale, of the resistance to 10^4^ Ohm at temperature of 550 °C occurred. From the profile of the curve in Figure 9, we can conclude that the conductivity was intrinsic in this temperature range. From the temperature dependence of the resistance, we can assume that in this case we are dealing with an electronic type of semiconductor [55,56,57]. In fact, the intrinsic concentration of charge carriers in an intrinsic semiconductor depends on temperature. In an n-type semiconductor, the number of free electrons (*n*) does not change significantly with increasing temperature, but the number of holes (*p*) increases [58]. However, in a p-type semiconductor, the number of free electrons (*n*) increases with temperature, but the number of holes remains constant [59].

**Figure 10:**
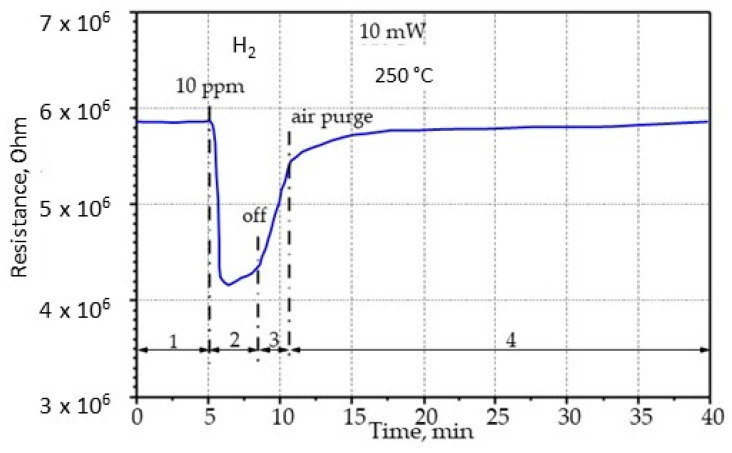
Chemical response of the thin-film sensor on an AA membrane to 10 ppm hydrogen at a temperature of 250 °C.

To understand the mechanism of gas adsorption on the nanostructured multicomponent film of the metal oxides, let us examine in detail the reaction sequence of the sensor using hydrogen as the sensing gas. Figure 10 shows the kinetics of adsorption of 10 ppm of hydrogen onto the gas sensitive layer of the sensor at a heating power of 10 mW. The curve shows four periods, as shown in Figure 10. During the first period, in the initial state of the sensor, when the purified air passed through the system, the resistance on the measuring electrodes was 5.8 MOhm. At the beginning of the second period, at the fifth minute, the hydrogen supply of 10 ppm started, due to chemisorption, the resistance of the gas-sensitive layer decreased sharply from 5.8×10^6^ to 4.2×10^6^ Ohm; then, it increased slowly for 3 min. Then the hydrogen supply was switched off, and the sensor resistance rose sharply to 5.5×10^6^ Ohm. The total response and recovery time was approximately 5 min. During the next 30 min, the system was purged with air, while within 5 min the sensor resistance was restored to its original value and did not change any more, which is shown in the curve as a plateau.

**Figure 11:**
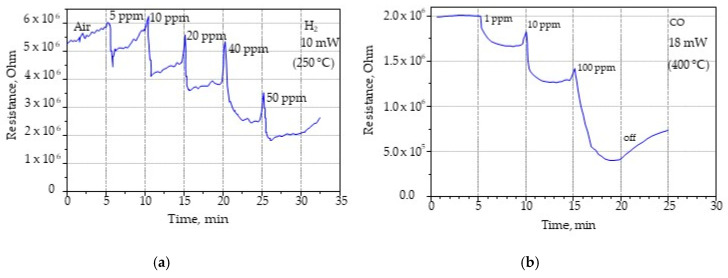
Responses of the gas sensor based on the thin membrane of nanoporous alumina substrate with a sensitive layer of Sn_x_Bi_k_Mo_y_O_z_: (**a**) for different concentrations of H_2_ at heating power of 10 mW (250 °C); (**b**) for different concentrations of CO at heating power of 18 mW (400 °C).

Since recovery of the sensor occurred quickly after the supply of test gas was stopped, and subsequent purging had virtually no effect on the recovery rate, we measured the sensor responses at different concentrations of H_2_ and CO, and the results at temperatures with better sensitivity to these gases are shown in Figure 11. Figure 11a shows the sensor responses at different hydrogen concentrations of 5, 10, 20, 40, and 50 ppm in the air-hydrogen mixture, without the sensor purge period between concentration changes. As can be seen in Figure 11a, the sensor responded almost immediately to different gas concentrations, while in five minutes, it had time to respond to a new gas concentration and recover.

The study of the gas sensitivity of the prepared thin film sensor was also carried out for CO at concentrations of 1, 10, and 100 pm (Figure 11b).

In the air-hydrogen mixture, the maximum response values were observed in the power range of 10–12 mW (250–300 °C). The response (named also sensitivity) of the Sn_x_Bi_k_Mo_y_O_z_ composite, calculated as the ratio of (R_air_–R_gas_)/R_air_, at 5 and 40 ppm of H_2_ concentrations at 250 °C was 0.22 and 0.40, respectively. As the temperature increased, the sensitivity of the sensor decreased due to the decrease in the sorption properties of the oxides. In this case, this occurred due to the sorption of the gas from the diode and a decrease in the resistance due to the exothermic reaction of hydrogen oxidation on the platinum electrodes of the heated sensor. Therefore, in percentage terms, as the temperature of the sensor increased due to the increase in power supplied to the heater, the exothermic reaction effect contributed less to the increase in sensitivity of the sensor. Such an effect at high temperatures of the gas sensitive layer may be because at such concentration values the sensor was already saturated.

The sensor maximum sensitivity to CO was obtained for a sensor power of 18 mW. This power value was 10 times lower than the power consumption of a sensor manufactured by traditional silicon MEMS technology [28,29,30]. For concentrations of 1 ppm and 10 ppm of CO, the sensitivity was shown to be 0.16 and 0.45, respectively. The gas-sensitive layer is a mixture of oxide with a low basic character from the homologous oxide series. In the temperature range corresponding to the maximum response, chemisorption mechanisms with formation of donor centers and partial reduction of higher oxides to lower oxides are possible. The gap transitions of tin and bismuth cause the increase in electron concentration in the conduction band.

The reproducibility of the measurements and the stability of the gas sensor systems were evaluated, and the results are presented in Table 1. 

The results were the average of measurements from at least five samples, with measurements repeated several times (10 times) over five days. The experimental results showed good reproducibility of the measurements and good stability of the sensors prepared for a few days with multiple use each day.

## 4. Conclusions

It has been shown that nanoporous substrates represent a perspective for the development of efficient sensors. The integration of heating elements and gas-sensitive layers on thin membranes of nanoporous AA profiled substrates were the novel steps introduced by our approach in the design of this type of sensor. A technological route was developed for the fabrication of modified AA profile substrates with locally deposited thin membranes. The design of the thin film sensor with the three-section meander heater and the interdigital counter was developed and experimentally tested in this work. The geometric dimensions of the structural elements were optimized according to the thermomechanical and gas sensitivity characteristics of the sensor. According to the developed technology path, in line with the design solutions, the sensor structures were fabricated on profiled AA substrates. A three-component nanocomposite based on Sn-O/Bi-O/Mo-O metal oxides, obtained by ion layering on the measuring elements, was used as gas-sensitive layers. The composition and properties of the metal oxide layers were investigated. The Sn_x_Bi_k_Mo_y_O_z_ nanocomposite forming the gas sensitive layer was identified as a highly resistive electronic-type semiconductor, whose resistance decreased from 10^6^ Ohm to 10^4^ Ohm when the temperature increased up to 550 °C. The experimental studies carried out showed that the prepared sensors were highly sensitive to hydrogen at a temperature of 250 °C, which was provided by the heating element at a power of 10 mW. The maximum sensitivity to carbon monoxide was achieved at a heating power of 18 mW, which allowed the temperature of the gas-sensitive layer to be raised to 400 °C. This value of power is less in 10 times then the power of a sensor fabricated by the MEMS technology. The presented results show real prospects for the design of microsystems for gas detection, using thin films on nanoporous profiled substrates and requiring low power. These microsystems consist of several micropowered sensors that should allow the detection of low concentrations of several flammable or toxic gases.

## Figures and Tables

**Table 1:** Data of the measurements of the sensing layer resistance in air and in loaded gas (H_2_ (10 ppm) and CO (10 ppm)).

Measurement Days	Resistance (MΩ) of Sensing Layer in Air at 250 °C	Resistance (MΩ) Measured in 1 min after Gas Load H_2_ (10 ppm)	Resistance (MΩ) of Sensing Layer in Air at 400 °C	Resistance (MΩ) Measured in 1 min after Gas Load CO (10 ppm)	Resistance (MΩ) Measured in 3 min after Gas Load CO (10 ppm)
Day 1	5.96 ± 0.20	4.29 ± 0.19	1.81 ± 0.13	1.22 ± 0.010	1.69 ± 0.10
Day 2	6.05 ± 0.25	4.52 ± 0.27	1.67 ± 0.17	1.12 ± 0.11	1.78 ± 0.08
Day 3	5.63 ± 0.21	4.39 ± 0.18	1.95 ± 0.15	1.3 ± 0.011	1.74 ± 0.07
Day 4	5.75 ± 0.14	4.01 ± 0.16	1.74 ± 0.08	1.18 ± 0.08	1.59 ± 0.10
Day 5	5.83 ± 0.09	4.15 ± 0.10	1.83 ± 0.11	1.19 ± 0.08	1.64 ± 0.04

## Data Availability

Not applicable.
